# Macrophage’s role in solid tumors: two edges of a sword

**DOI:** 10.1186/s12935-023-02999-3

**Published:** 2023-07-31

**Authors:** Arian Jahandideh, Mahsa Yarizadeh, Maryam Noei-Khesht Masjedi, Mina Fatehnejad, Romina Jahandideh, Roben Soheili, Yeganeh Eslami, Maryam Zokaei, Ardavan Ahmadvand, Nogol Ghalamkarpour, Rajan Kumar Pandey, Mohsen Nabi Afjadi, Zahra payandeh

**Affiliations:** 1 Student Research Committee, Mazandaran University of Medical Sciences, Sari, Iran; 2 Usern Office, Mazandaran University of Medical Sciences, Sari, Iran; 3 Islamic Azad University, Tehran Medical Branch, Tehran, Iran; 4 Department of Medical Biotechnology, School of Advanced Technologies in Medicine, Tehran University of Medical Sciences, Tehran, Iran; 5 Faculty of Medicine, Tehran University of Medical Sciences, Tehran, Iran; 6 Department of Microbiology, Faculty of Advanced Science and Technology, Tehran Medical Science, Islamic Azad University, Tehran, Iran; 7 Faculty of Medicine, Mazandaran University of Medical Sciences, Sari, Iran; 8 Department of Food Science and Technology, Faculty of Nutrition Science, Food Science and Technology/National Nutrition and Food Technology Research Institute, Shahid Beheshti University of Medical Sciences, Tehran, Iran; 9 Faculty of Medicine, Iran University of Medical Sciences, Tehran, Iran; 10 Department of Clinical Laboratory Sciences, School of Allied Medicine, Student Research Committee, Lorestan University of Medical Sciences, Khorramabad, Iran; 11 Department Medical Biochemistry and Biophysics, Division Medical Inflammation Research, Karolinska Institute, Stockholm, Sweden; 12 Department of Biochemistry, Faculty of Biological Sciences, Tarbiat Modares University, Tehran, Iran

**Keywords:** Cancer, Macrophage, Glioma, Melanoma, Tumor

## Abstract

The tumor microenvironment is overwhelmingly dictated by macrophages, intimately affiliated with tumors, exercising pivotal roles in multiple processes, including angiogenesis, extracellular matrix reconfiguration, cellular proliferation, metastasis, and immunosuppression. They further exhibit resilience to chemotherapy and immunotherapy via meticulous checkpoint blockades. When appropriately stimulated, macrophages can morph into a potent bidirectional component of the immune system, engulfing malignant cells and annihilating them with cytotoxic substances, thus rendering them intriguing candidates for therapeutic targets. As myelomonocytic cells relentlessly amass within tumor tissues, macrophages rise as prime contenders for cell therapy upon the development of chimeric antigen receptor effector cells. Given the significant incidence of macrophage infiltration correlated with an unfavorable prognosis and heightened resistance to chemotherapy in solid tumors, we delve into the intricate role of macrophages in cancer propagation and their promising potential in confronting four formidable cancer variants—namely, melanoma, colon, glioma, and breast cancers.

## Introduction

Cancer immunotherapy has become a potent disease treatment option that helps advanced cancer patients survive longer while removing any chance of returning tumors [1, 2]. In cancer patients, immune cells are ineffective against cancer cells and promote tumor growth, decreasing treatment effectiveness [3, 4]. Among innate system cells, macrophages play a crucial role in normal homeostasis, inflammation, and phagocytosis [5, 6]. However, macrophages have been shown to play a negative role in the progression of oncogenesis and neoplastic disease by promoting genetic instability and angiogenesis. [7]. Macrophages are divided into the M1 and M2 subgroups based on morphological, phenotypic, and functional variability. The M2 macrophages have been shown to support tumor growth and metastasis, whereas the M1 macrophages play a crucial role in antitumor immunity and largely orchestrate pro-inflammatory activities in the tumor microenvironment (TME) [8, 9]. Tumor-associated macrophages (TAMs), the most diversified immune cells in the TME that are essential for tumor formation, include the M2 macrophages and a small population of M1 macrophages [10]. In this line, tumor cells secrete chemokines and growth factors to draw in macrophages and change them into the M2 type. Therefore, it was also discovered that significant dynamic changes in macrophage subpopulations were related to the efficacy of immunotherapy [11]. Therefore, this critique outlines the latest developments in the functions of TAMs in predicting, detecting, and treating four potent forms of cancer, namely melanoma, colon, glioma, and breast, which have been extensively explored in previous research publications.

## Macrophage-based therapy for breast cancer

Breast cancer includes four main groups: inflammatory breast cancer (IBC), ductal carcinoma in situ (DCIS), invasive ductal carcinoma (IDC), and metastatic breast cancer [12]. The IDC is particularly aggressive and responsible for most breast cancer cases. Since breast cancer cells become resistant to traditional cancer treatments, researchers are seeking new approaches, such as TAMs therapy, which involves macrophages targeting the tumors to control their growth and spread.

### TAMs promote cell stemness in breast cancer

The tumor microenvironment (TME), which includes the surrounding cells and molecules that interact with cancer cells, plays a critical role in the development of tumors, including the occurrence, progression, and immune suppression of the tumors [13]. Molecules existing in the tumor microenvironment, commonly upregulated in the tumor stroma, have been shown to influence the behavior of macrophages and their ability to infiltrate and polarize within the tumor microenvironment [14]. So, the specific role of TAMs in cancer prognosis may vary depending on the cancer types and their ability to adapt in response to the tumor microenvironment [15]. The TAM can exhibit both M1-like pro-inflammatory and M2-like immunosuppressive traits, such as the production of anti-tumor-molecules as well as T cells priming, and secretion of immunosuppressive molecules and expression of inhibitory checkpoint proteins, respectively [16]. In breast cancer, TAM may exhibit a combination of M1- and M2-like traits contributing to the formation of pre-metastatic niches [17] and pro-angiogenic processes [18]. Moreover, various literature evidence suggests that many immunosuppressive cells within the TME play a significant role in the maintenance and expansion of cancer stem cells (CSCs) [19]. This is due to the ability of TAMs to produce various cytokines promoting the self-renewal and proliferation of CSCs. For example, M1 macrophages can also stimulate the formation of drug-resistant ALDH1^+^ breast CSCs [20]. Additionally, the TAMs have been found to secrete IL-6, which can stimulate the transformation of non-stem cancer cells into CSCs by activating the JAK/STAT pathway [21]. Furthermore, the TAMs can upregulate the expression of SOX transcription factors and surface receptors, which can enhance the CSC phenotype in breast cancer cells by activating the EGFR/STAT3/SOX-2 pathway [22]. The expression of SOX-2 in early-stage breast tumors is important in the regulation of CSC formation [23]. Finally, during epithelial–mesenchymal transition (EMT), the upregulation of EPHA4 on the TAMs surface can bind to cancer cells receptor, leading to activating the NF-kB pathway to facilitate the maintenance of homeostasis in CSCs [24].

### TAMs induce immunosuppressive microenvironment in breast cancer

**Fig. 1:**
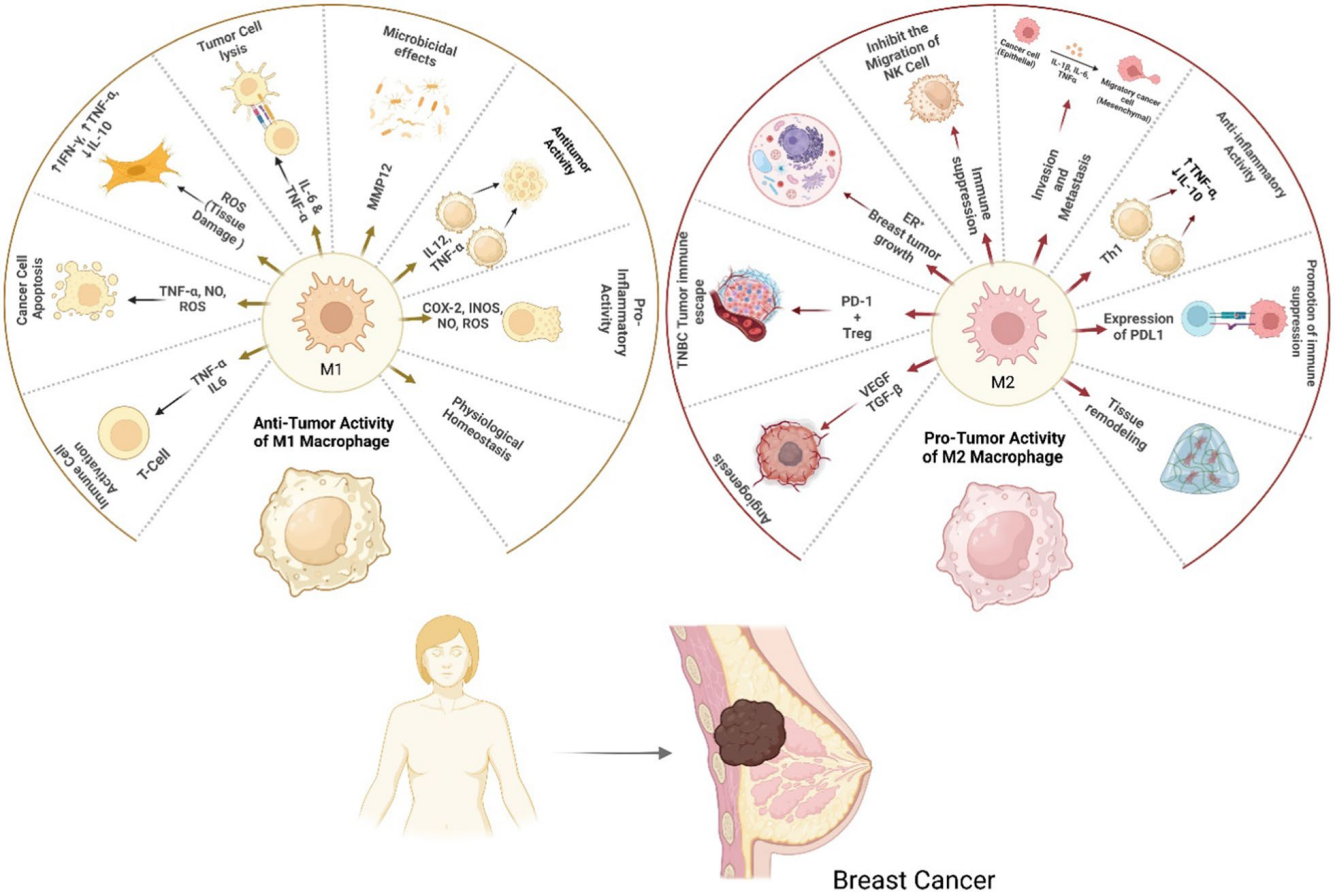
Anti-tumor/pro-tumor activity of macrophages in breast cancer

TAMs are also found in the TME, playing a significant role in cancer progression (Fig. 1). Their main function is to regulate the T cell's function, specifically effector T cells, to kill cancer cells [25]. This is achieved through various mechanisms, including regulating arginine metabolism, producing nitric oxide, and expressing immune checkpoints such as programmed cell death protein-1 (PD-1) [26]. TAM-secreted IFN-γ activates the JAK/STAT3 and PI3K/AKT pathways to increase PD-L1 expression while transforming growth factor beta (TGF-β) polarizes macrophages towards an M2 phenotype, which enhances the suppressive activity of TAMs, upregulates PD-L1 expression and facilitates tumor escape. Additionally, PD-L1 expression is considerably upregulated in the absence of IL-6 and has been found to be highly effective when treated with an anti-PD-L1 antibody [27, 28].

TAMs can also exhaust CD8+ T cells and reduce their ability to eliminate cancer cells [29]. So, understanding its mechanisms is a key area of focus in developing cancer immunotherapies. A TNBC-based study was conducted by Xu and colleagues using single-cell transcriptome analysis to examine the relation between TAMs and exhausted T cells [30]. Moreover, the secretion of STAT3 by TAMs into the TME, with their increasing numbers in the stroma, can lead to CD8+ T cell exhaustion [31]. TAMs and myeloid-derived suppressor cells can also suppress immune function through cell-to-cell contact, stimulating myeloid-derived suppressor cells (MDSCs) to secrete IL-10 and inhibit IL-12 production via dendritic cells [32]. The TAMs also play a role in inhibiting T cell recruitment, so targeting certain pathways, such as colony-stimulating factor-1 (CSF1/CSF1R) [3], can obstruct macrophage recruitment and promote T cell infiltration [33].

### TAMs targeting breast cancer therapy

Currently, CSF-1R is inhibited by PLX3397 to diminish M2 macrophage recruitment, which is utilized to treat malignancies such as glioblastoma, breast cancer, and other tumors. There was high tolerability in a phase 1 study of the CSF-1R inhibitor LY3022855 in metastatic breast cancer [34]. Twenty-two medicines that target CXCR4 are now in the active development phase; most of these are small molecule antagonists; however, there are also antibody-based medications, gene therapies, and CAR-T cell treatments. Eighteen of these medications are being developed to treat solid tumors and hematological malignancies. Mozobil (Plerixafo), a small molecule antagonist that targets CXCR4, was introduced in 2018. It is first utilized with granulocyte colony-stimulating factor (G-CSF) to provoke hematopoietic stem cells for therapy of multiple myeloma and non-lymphoma Hodgkin’s.

To evaluate IMM2902’s safety and effectiveness in HER2+ advanced solid tumors, clinical trials have approved the drug's primary indication of the lung, gastric HER2-positive breast, and other solid tumors (NCT05076591). SIGLEC10 interacts with CD24 in renal clear cell carcinoma, triple-negative breast, and ovarian cancers to prevent tumor cell phagocytosis and immune cell activation. Blocking SIGLEC10hi TAMs in HCC decreased the expression of immunosuppressive molecules and increased the cytotoxic effects of CD8+ T cells. It also supported Pembrolizumab as an anti-tumor drug that targets PD-1 molecules [35]. Additionally, anti-MARCO therapy reduced the metastasis and development of mouse melanoma and breast cancer, improved the TME's immunogenicity, and increased the treatment effectiveness of anti-CTLA4 mAbs [36, 37].

#### Repolarization of TAMs into M1-type macrophages exerts tumor-killing effects

**Fig. 2:**
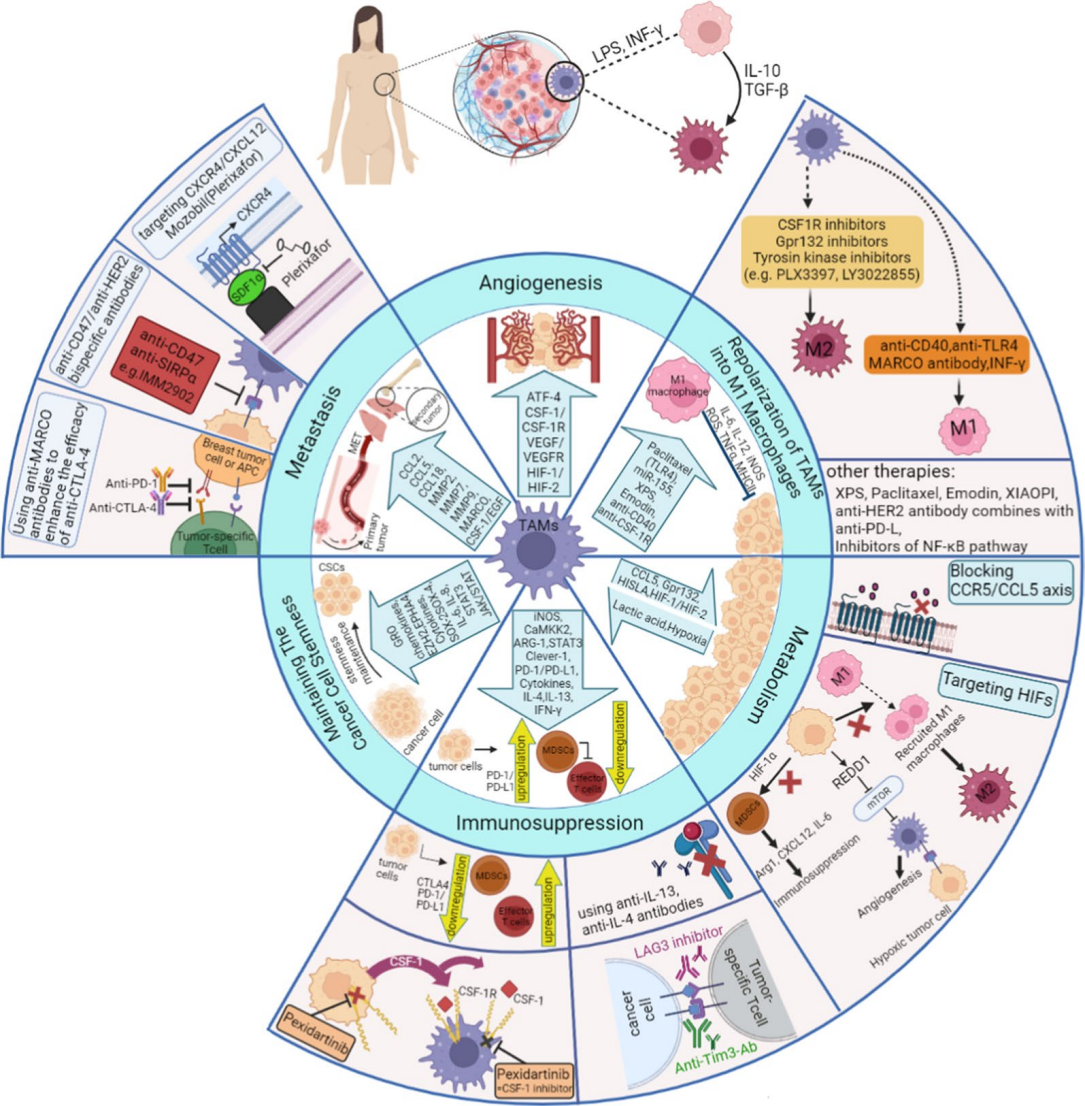
Different macrophages-based treatment strategies in breast cancer

The alteration of TME leads to the polarization of TAMs into M1 macrophages, facilitating an immune response against the tumor. The M1 macrophages exhibit a strong antigen presentation ability as they express major histocompatibility complex class II and secrete various proinflammatory cytokines, including interleukin-6, interleukin-12, inducible nitric oxide synthase, reactive oxygen species, and tumor necrosis factor-alpha (TNFα), which have the potential effect of killing cancer cells [38]. So, repolarizing TAMS into M1 macrophages leads to increased secretion of interleukin-12, which activates and recruits natural killer cells to carry out tumor cell killing in advanced tumors [39]. Additionally, the use of anti-Her2 antibodies in combination with anti-PD-L is beneficial in upregulating PD-L1 expression in macrophages [40]. Traditional Chinese medicine may also serve as a potential therapeutic option for breast cancer by promoting the repolarization of TAMs, with emodin and the XIAOPI formula being particularly effective in this regard. Emodin, a Chinese herbal medicine, exerts anti-tumorigenic impacts on breast cancer by suppressing the production of transforming growth factor beta 1 (TGFβ1) in macrophages [41]. The key bioactive compound of the XIAOPI formula, Baohuoside I, has also been shown to block the polarization of TAMs’ M2 phenotype and severely restrict the invasion and migration of breast cancer cells [42]. Various macrophage functions and different treatment options for breast cancer are shown in Fig. 2.

## Macrophages-based therapy in colorectal cancer

Colorectal cancer (CRC) is the third most prevalent cancer type and in terms of mortality ranks the second leading cause of cancer deaths [43]. A variable antitumor immune response gets elicited in colorectal malignancies [44], and high tumor-associated T-cell concentrations are recognized as positive prognostic indicators [45, 46]. As a result, the immune system may also contribute to tumor progression [47, 48], which will be discussed below.

### Role of TAMs in colorectal cancer (CRC)

**Fig. 3:**
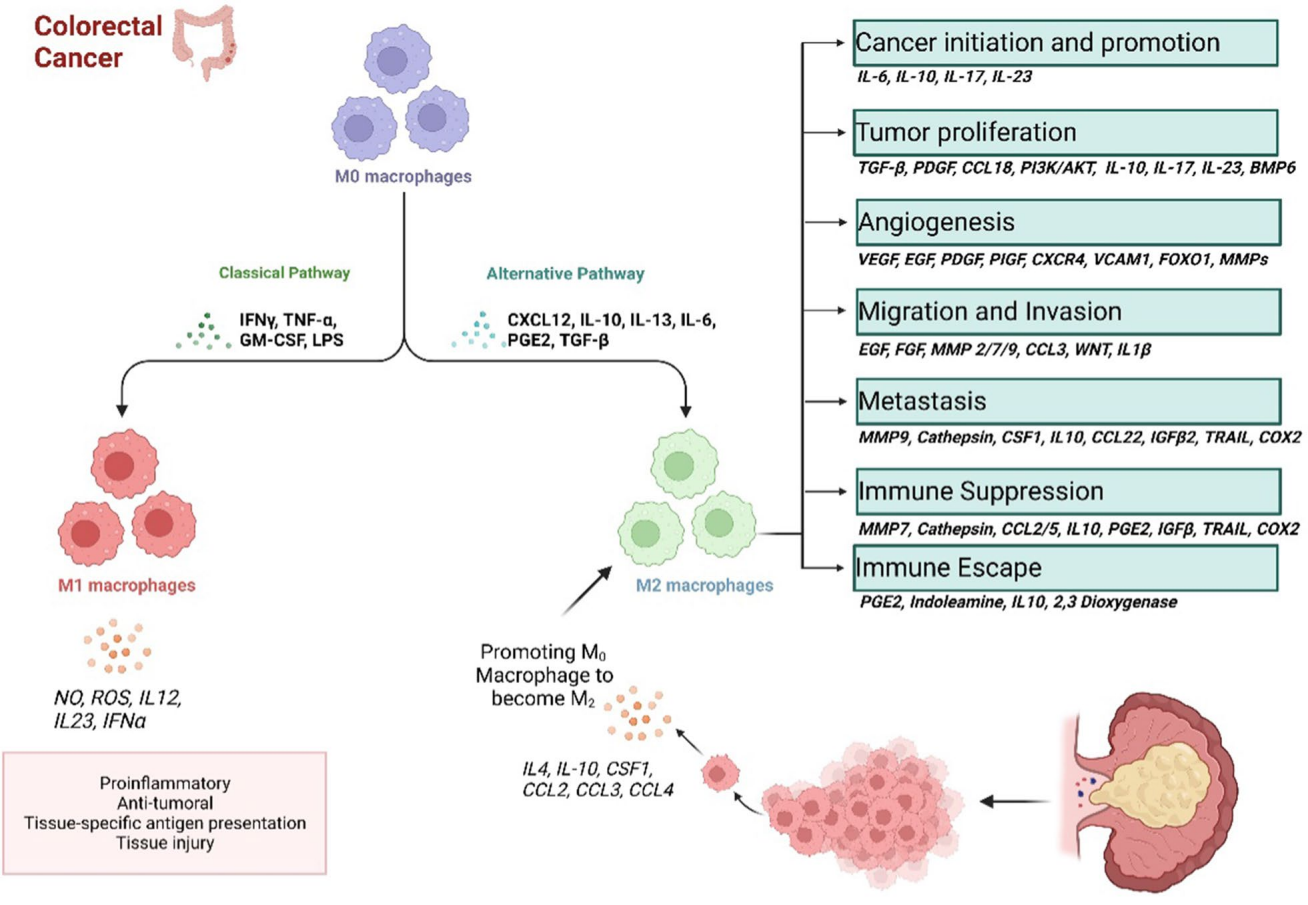
Anti-tumor/pro-tumor activity of macrophages in CRC

Most studies demonstrate the relationship between macrophage infiltration and the clinical course of CRC. Some studies have found a correlation between higher macrophage infiltration, more advanced tumor stages [44], and worse prognosis [49]. In contrast, other studies indicate that TAMs can improve the prognosis and progression of CRC (Fig. 3) [50]. These discrepancies may be due to the use of CD68, a macrophage lineage marker, without considering differences among the various anti- or pro- inflammatory subtypes [51]. A meta-analysis found that a high density of CD68 macrophages in the tumor microenvironment was associated with a better prognosis and a lack of tumor metastasis in CRC patients [52]. This may be due to the recruitment of macrophages contributing to an adaptive immune response against the tumor [53–55]. Additionally, a high density of CD68-labeled macrophages in the tumor microenvironment was correlated with high infiltration of CD8 T cells and CD3 T cells, which can regulate the macrophage polarization to the M2 subtype leading to CRC metastasis. Recent research has analyzed markers for M1 and M2 subtypes. They found that in the early stages of the CRC, there was a correlation between macrophage infiltration and enhanced disease-free survival, while in later stages of the disease, a high number of CLEVER-1/Stabilin-11+ cells, an M2 marker, correlated with shorter disease-free survival [56].

### The role of TAMs on CRC angiogenesis

Evidence shows that the number of TAMs in a tumor is related to the number of blood vessels in malignancies [57–60]. The TAMs are recruited to hypoxic areas of the tumor, where they secrete various molecules that promote angiogenesis and provide nutrition for tumor growth. The HIF-1α, expressed in TAMs and other cells, regulates the transcription of genes associated with angiogenesis in hypoxic sites in a HIF-1α-dependent manner [61]. Several studies have found a correlation between macrophage infiltration and vascular density in colorectal cancer [61, 62]. These studies suggested that TAMs are the key regulators of tumor angiogenesis in this type of cancer [62]. The GPR35, expressed on macrophages, has been shown to promote tumor angiogenesis and MMP activity by activating Na/K-ATPase. Macrophages also secrete MMP-2 and MMP-9, enzymes that improve cancer angiogenesis in vivo, under the stimulation of L-10 [63–65]. Additionally, MK2 signaling and angiogenesis are inherent in macrophages [66]. Through modulating NADPH oxidase activity, TAMs can also improve angiogenic protein expression in the tumor microenvironment [67]. In this line, chronic inflammation in the sub-epithelial stroma is hypothesized to activate mutagenic mechanisms that initiate tumor formation [68]. The pro-inflammatory M1 macrophages [69] can potentiate this effect by triggering oncogenic mutations in the adjacent epithelial layer [70]. Colon cancer cells produce macrophage colony-stimulating factor-1 to attract and re-educate macrophages. Tumor development in its early stages attracts monocytes and ensures their maturation into macrophages within the tumor microenvironment [71]. Later, these cells differentiate into TAMs, and cancer cells manipulate their metabolism through different signaling pathways to support further tumor growth and progression. Chemoattractant CCL2 overexpression has been linked to advanced tumor stages, metastatic disease conditions, and poor prognosis in CRC. Furthermore, CRC cells produce lactic acid through aerobic glycolysis as a byproduct [72]. Even oxygen does not hinder this process; they metabolize glucose to lactate, which induces the expression of arginase-1 and VEGF. This mechanism recruits and polarizes macrophages towards the M2 phenotype and helps in tumor-promoting, therefore associated with TAMs metabolic reprogramming [67]. Lim, S.Y. et al. reported TAMs-mediated S100A8/A9 mRNA expression in colon cancer involving ERK-signaling and inducing tumor cell migration [73]. Phinney et al. also reported TAMs-mediated secretion of chemokines such as monocyte chemotactic protein-1 (MCP1) and macrophage inflammatory proteins-1 and -2 (MIP-1 and MIP-2) by the use of the MAPK-activated protein kinase 2 (MK2) pathway, helping tumor cell growth and invasion in vitro [74]. Wei, C. et al. showed that IL-6 secretion by TAMs stimulates EMT, thereby improving CRC invasion and migration ability through the modulation of the JAK2/STAT3/miR-506-3p/FoxQ1 axis [75]. β-catenin nuclear localization promoted by STAT3 improves growth regulation [76]. In addition, HT29 and HCT116CRC cells increased vimentin expression level although decreased E-cadherin level, indicating improved invasion ability [77]. The TAMs also transfer microRNAs to cancer cells, leading to the downregulation of proteins that inhibit metastasis and the upregulation of proteins that promote it [78]. Overall, these findings highlight the complex role of TAMs in CRC development and progression [79].

### TAM's roles in the prognosis of CRC

The macrophage polarization state was a predictive factor independent of common tumor molecular and clinical characteristics. On the other hand, several studies suggest that CD68+ TAMs, which are primarily found in the stroma of CRC along the front edge of invasion, are associated with better prognoses for CRC patients [51, 80, 81]. For instance, in a 30-patient Japanese CRC cohort, a lower density of CD68+ in the tumor stroma and invasive front were linked to more progressive cancer, whereas high levels of TAMs were linked to a favorable prognosis [82]. Similar relationships got observed in European cohorts. For instance, in a tissue microarray of 100 colon cancer patients in Germany demonstrated decreased CD68+ macrophages in higher-stage tumors [83]. In a Bulgarian cohort conducted on 210 patients with primary CRC, a lower density of CD68+ TAMs in the invasive tumor front which is considerably associated with the advanced tumor stage (III and IV stages), distant metastases, and local lymph nodes specific metastases was observed [84]. A lesser number of CD68^+^ TAMs were also reported in cancer patients where the tumor cells migrated and invaded the blood circulation, lymph vessels, and perineural tissues.

Additionally, a high CD206/CD68 ratio has been linked to improved recurrence-free survival rates in patients with stage II of CRC after receiving adjuvant chemotherapy [85]. On the other hand, both VEGF-expressing and CD68-TAMs have been found to predict improved survival rates in individuals with stages II and III of CRC. So, only TAM infiltration cannot fully explain the degree of disease recurrence. A recent meta-analysis of 6115 CRC cases from 27 separate studies indicated a high density of TAMs in CRC as an independent favorable predictor for 5-year OS but not for DFS. The TAM density and additional prognostic markers may be a more accurate predictor of CRC relapse. In this line, traditional methods of analyzing TAMs have relied on the expression of CD68, a pan-macrophage marker, but recent studies have used double immunofluorescence staining to identify different subsets of TAMs using other markers such as CD86, CD163, and CD206 [86, 87]. Literature has shown that the presence of M2 macrophages (CD163^+^) is correlated with poorer overall survival and disease-free survival/recurrence-free survival in CRC. In addition, high levels of CD163^+^ TAMs and a high CD163/CD68 ratio have been linked to an aggressive phenotype and poor prognosis in CRC [77]. In a recent study, as the proportion of M2 TAMs in total TAMs, co-expression of CD68 and CD163 was found to be a better predictor of prognosis than traditional clinic pathological factors or the expression of CD163 TAMs alone [80]. Additionally, the expression of CD86 TAMs and TNM stage were found to be independent prognostic factors for recurrence-free survival and overall survival in CRC [88].

### Potential applications of TAMs in CRC therapy

#### Blocking monocyte infiltration in CRC

Blocking the infiltration of mononuclear cells, such as TAMs, in the inflammatory tissues associated with tumors has been identified as a potential therapeutic method for primary cancers. Chanmee et al. demonstrated that TAMs, specifically those associated with colon cancer, induce CXCR4, CXCL-12, and HIF-1 in the hypoxic TME. Moreover, the accumulation of TAMs is blocked by targeting the HIF-1/CXCR4 axis effectively. Mantovani et al. revealed that TAMs derived from colon cancer monocytes could differentiate, highlighting the need for combination therapies that block differentiation to target these cells effectively. In this line, the TNF-α has been found to induce the recruitment of monocytes and simultaneously inhibit the differentiation of monocytes or macrophages into TAMs in the TME of colon cancer in vivo [89]. Another strategy for targeting TAMs is the inhibition of their recruitment or infiltration. SIX1, a protein that is overexpressed in various types of cancer and promotes the recruitment of pro-tumor TAMs to the region of colorectal cancer (CRC) [79], can be silenced through the use of its inhibitor, Nitazoxanide, which suppresses the WNT/CTNNB1 pathway [90, 91]. Trifluridine/Tipiracil, an anti-metabolism drug, has been observed to effectively exhaust M2 macrophages when combined with oxaliplatin, leading to the infiltration of cytotoxic CD8+ T cells and the lysis of tumor cells [92].

#### Repolarizing TAMs

TAMs predominantly exhibit an M2 phenotype, simultaneously promoting immunosuppression and angiogenesis. They can be re-educated via M2 to M1 polarization. For instance, TAMs mediated inhibition of macrophage receptor expression with collagenous structure (MARCO) repolarized TAMs to the M1 phenotype and caused antitumor activity in the MC38 colon cancer mice model [93]. By altering the number and frequency of myeloid cells infiltrating the tumor, tasquinimod-based immunotherapy can reduce the immunosuppressive potential of TME [94]. It has been demonstrated by Olsson et al. that tasquinimod targets early-stage myeloid cells that tend to penetrate tumors, causing M2 myeloid cells to adopt an M1 macrophage phenotype, altering the tumor microenvironment, preventing angiogenesis, and inhibiting metastatic spread [95].CRC can be diagnosed and treated using long non-coding RNAs (lncRNA) as noninvasive biomarkers and targeted molecules. For instance, cells secreting lncRNAs, such as RPPH1, promote M2 polarization and tumor metastasis but can't be directly targeted [90, 91]. Cathepsin K (CTSK), which binds to TLR4 and activates mTOR, is synthesized by intestinal microflora and modulates the expression of long noncoding RNAs in various tissues [92]. The CTSK-specific inhibitor Odanacatib has been reported to curb the pro-tumor effects and improve the prognosis of CRC patients [96]. Moreover, researchers found that Ru@ICG-BLZ nanoparticles effectively repolarize TAMs to M1 macrophages due to their CRC specificity and low toxic properties as a new approach [97].

#### Targeting TAMs in immunotherapy

Immune checkpoint inhibitors, T cells-based treatment, and autologous tumor vaccines are the key components of immunotherapy in CRC treatment [98–101]. These strategies target immune checkpoint inhibitors with matching targets, including CTLA-4, PD-1, and PD-L1 [3, 10, 102]. The co-inhibitory molecule CTLA-4, produced by T cells, binds to the ligand CD80/86 on adenomatous polyposis coli (APC) to produce an inhibitory signal [103]. When PD-1, an immunosuppressive receptor on T cells, binds to PD-L1, it significantly reduces the activity of antigen-specific T cells [104]. PD-L1 is expressed mainly by aggressive primary tumor cells and by CD68/CD163-positive M2 macrophages in patients with colorectal cancer with high microsatellite instability [105]. Gordon and his team discovered that when the illness worsens, TAMs express more PD-1. Additional research revealed the ability of TAMs to phagocytose is inversely linked with PD-1 expression, and in vivo*,* inhibition of PD-1-PD-L1 improved macrophage phagocytosis, slowed tumor growth, and lengthened mouse survival [16]. On the other hand, evidence suggests that the PD-1/PD-L1 axis plays a role in skewing TAMs from the M1 to M2 phenotype, and M2 TAMs have been found to contribute to resistance to PD-1/PD-L1 blockade [106, 107]. As such, switching TAMs from the M2 to the M1 phenotype is a promising strategy for improving the efficacy of checkpoint blockade therapy, and strategies aimed at “re-educating” TAMs are being developed to overcome the current limitations of immunotherapy [108]. In conclusion, the relationships between PD-1/PD-L1 expression and polarization in TAMs seem to be crucial in tumor progression, indicating that combination immunotherapy targeting these cells will likely become a trend in checkpoint blockade therapy [109].

**Fig. 4:**
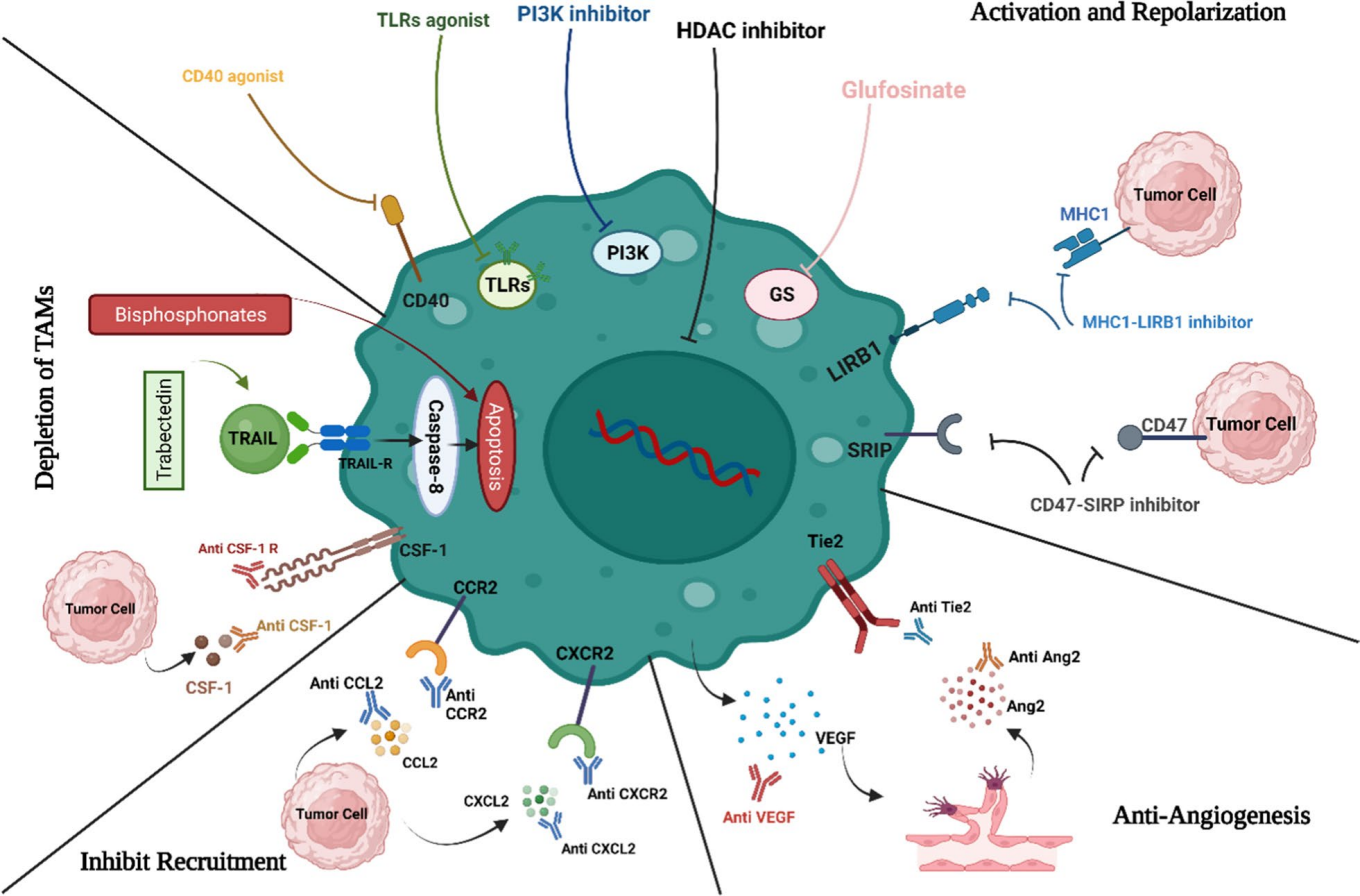
Macrophages-based therapeutic strategies in CRC

So, PDCD1 blockers can increase macrophages’ capacity to phagocytose and lengthen patient lifetime, supporting the idea that TAM can be the target of PDCD1 therapy. Additionally, patients with higher M2 macrophage infiltration in lesion sites may benefit from increased efficacy. Regarding adoptive cell treatment, tumor-directed anti-mesothelin CAR-T cells and M2 inhibitors have been shown to have anti-tumor efficacy. In contrast, CD40-based TAM-associated adoptive cell therapy is currently being researched [110]. The OVA vaccine may lessen the density of TAMs in cell models that persistently express ovalbumin (OVA) peptides. Additionally, administering a VEGFC/VEGFR3 neutralizing antibody may further block the chemotaxis of M2 macrophages into the CRC region, reducing tumor growth and preventing the CRC from evading immune surveillance (Fig. 4) [111].

## Tumor-associated macrophages in melanoma

In the intricate management of interactions, networks, and linkages between melanoma cells and other cell subpopulations that make tumor stroma, macrophages play a crucial role. Macrophages influence the tumor microenvironment by producing different proteins, enzymes, and oxidants, encouraging tumor development and invasion. In advanced primary melanoma lesions, it was found to have a decrease in the number of macrophages while an increase in the iNOS-positive macrophages. Markedly, the macrophage-produced nitric oxide has shown anticancer properties, but in the presence of INFγ producing NK cells [112]. The expression of cyclooxygenase-2 (COX-2), a pro-inflammatory protein, characterizes Melanoma-associated macrophages. Regarding percentage, the presence of COX2-positive TAMs is highest in thin melanoma and lowest in advanced metastatic melanomas. Therefore, COX-2 has been considered a melanoma progression marker [113]. Osteopontin-stimulated macrophages in the melanoma microenvironment begin to produce the COX-2 protein [114], where α9β1 integrin acts as an osteopontin-specific surface receptor on macrophages and stimulates COX-2 expression via ERK and p38 pathways [114]. In addition, to maintain an inflammatory environment, melanoma cell angiogenesis, and migration are supported by COX-2 through COX-2-dependent PGE2 production [114]. Renalase is the next protein that promotes melanoma growth in the tumor microenvironment. This flavoprotein functions as a cell survival factor found in CD163-positive TAMs and melanoma cells [115].

Melanoma-associated macrophages also indirectly promote angiogenesis by releasing TNF-α and IL-1α. Upon stimulation, melanoma cells generate various angiogenic factors such as VEGF, TIE2, IL-8, and CD31, leading to neoangiogenesis [116]. Additionally, pericytes released proangiogenic factor milk fat globule-epidermal growth factor 8 (MFG-E8) stimulates M2 macrophage polarization, suggesting their role in tumor angiogenesis. The tumor microenvironment is characterized by a hypoxic condition found to be associated with TAMs, and it also affects TAMs [117]. Tumor-specific hypoxic condition drives TAMs accumulation in the melanoma microenvironment [117]. It has been established that under hypoxic situations, melanoma cells release the high-mobility group box 1 (HMGB1) protein, which promotes the M2 macrophage accumulation and IL-10 production in the tumor microenvironment [117], activating advanced glycation end-product receptors and leading to an inflammatory response.

### Interactions of macrophages with melanoma cells

Literature reports on mice with spontaneous melanoma have shown that stem-like cells and CD34 tumor-initiating cells (TICs) depend on M2 macrophages for initiating tumor growth and determining specific tumor characteristics, including chemo-resistance. The proliferation and survival of TICs are also dependent on TAMs [118]. In addition, TICs get stimulated by TAMs to form melanospheres or non-adherent colonies of melanoma cells [118]. It has been indicated that CD34-TICs induction through TAMs leads to melanoma development [118]. These studies also revealed that chemotherapies, namely cisplatin and temozolomide, drive TAM recruitment in the tumor microenvironment, stimulate TAM-responsive TICs growth, and protect TICs against chemotherapy effects [118]. At the molecular level, TICs stimulation results from TAM-derived TGF-β, which regulates and stimulates arginase production, leading to the production of polyamines playing a key role in the growth and differentiation of cancer cells [119].

### Role of TAMs in melanoma therapy

The macrophage colony-stimulating factor (M-CSF) [120] and granulocyte-macrophage colony-stimulating factor (GM-CSF) can result in the induction of M2- and M1-like TAMs, respectively. So, blocking M-CSF receptors on MDSCs can lead to the preferential expression of M1 phenotypes [36]. The macrophage receptor with collagenous structure (MARCO) regulates PI3K/AKT/mTOR signaling pathways and M1 or M2-like TAM polarization [121–123]. Additionally, new targeted treatments for disseminated melanoma (using, for example, an anti-PD-L1 antibody) were made possible by our understanding of signaling pathways, protein molecules, and their ligands [124]. Durvalumab, atezolizumab, and avelumab are the anti-PD-L1 monoclonal antibodies used to treat melanoma. Atezolizumab (anti-PD-L1), an FDA-approved mAbs, was recommended for use in combination with the BRAF inhibitors cobimetinib and vemurafenib to treat metastatic melanoma that has the BRAF V600 mutation [125–128]. Two anti-CTLA-4 mAbs are ipilimumab and tremelimumab. Ipilimumab and nivolumab combination therapy for metastatic melanoma has also shown a 52% survival rate for 5 years [129].

Targeted medicines may be utilized to support the already used therapeutic approaches or directly target TAMs to eradicate them or control their activity. It has been discovered that macrophage activation causes them to become more active against melanoma [130]. Pathogen vaccination, nanoparticles (polyhydroxylated fullerenols), galectin-9, GM-CSF, and inhibiting melanoma suppression of macrophage movement by macrophage inhibitory cytokines are some immunomodulatory agents that can be used to achieve this goal [130]. Preventing macrophage conversion to TAMs is another therapeutic approach. Antibodies that block TGF-β, Il-4, or Il-10 may also be employed in this technique [130]. Finally, research is being done on TAM-targeted treatments [131]. Preclinical investigations of the STAT-3 inhibitor, Janus Kinase-2 inhibitor, or nanoparticles that carry small interfering RNA to TAMs are promising [132].

#### Targeting TAMs-derived chemokines

Stromal factors influence the chemokines produced by TAMs in skin malignancies, and they help characterize the profile of TILs in the tumor microenvironment. Periostin (POSTN) is produced in the lesions surrounding melanoma cell nests in metastatic melanoma. The TAMs are primarily present in the tumor stroma, and POSTN encourages CD163^+^ macrophages to release various cytokines, including Treg-related chemokines (CCL17 and CCL22) [133, 134]. Repolarizing TAMs by immunomodulators such as imiquimod and IFNs may inhibit melanoma tumor growth as TAMs generated by CCL17 and CCL22 attract Tregs to melanoma tumor locations [135, 136]. Certain chemokines, including IL-8, CCL4, CCL17, and CXCL10, in the cerebrospinal fluid may predict brain metastases in melanoma patients. The use of certain chemotherapy agents, such as nimustine hydrochloride, dacarbazine, and vincristine, has been shown to decrease CCL22 production in B16F10 melanoma mice [134]. These findings suggest that TAM-derived chemokines produced in the tumor stroma under the influence of POSTN (a protein involved in extracellular matrix organization) may contribute to melanoma-specific TILs in melanoma patients [137].

#### Targeting TAMs-derived angiogenic factors

TAMs have been shown to produce various angiogenic factors, including platelet-derived growth factor (PDGF), VEGF, TGFβ, and matrix metalloproteinases (MMPs) [138]. These factors can promote neovascularization by recruiting TAMs to the location of skin tumors in mouse models [139]. The VEGF and MMPs have been identified as crucial indicators of skin cancer progression [140], with high concentrations of POSTN and CD163^+^ TAMs in the tumor stroma of skin malignancies leading to increased production of MMP1 and MMP12 in skin lesions [141]. TAMs stimulated by tumor stromal factors may therefore serve as potential targets for molecular targeted therapy in the treatment of cancers [133].

#### Effects of anti-cancer agents on TAMs

Recent studies have also concentrated on the immunomodulatory impacts of chemotherapeutic drugs on TAMs. For instance, in mouse melanoma models, non-cytotoxic dosages of paclitaxel could reduce MDSCs and even prevent their ability to suppress the immune response [142]. It has been discovered that chemotherapeutic agents and drugs with low molecular weight co-localize along with TAMs at tumor locations. Hu-Lieskovan et al. showed combination therapy with dabrafenib and trametinib with synergistic impacts of immune checkpoint inhibitors. In contrast, dabrafenib and trametinib monotherapy led to elevated Tregs and decreased TAMs in melanoma, respectively [143].

**Fig. 5:**
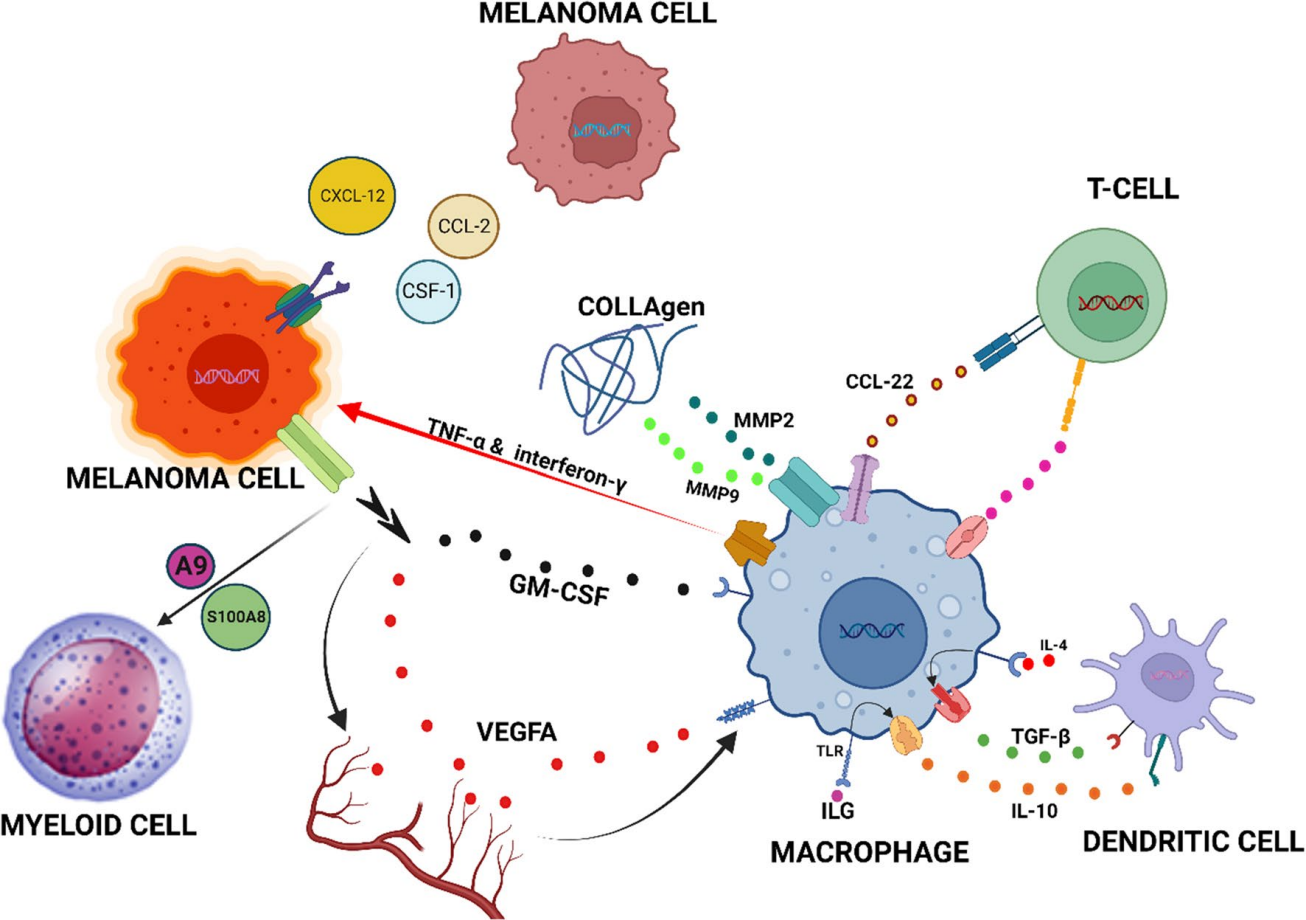
Role of TAMs in melanoma occurrence and therapy. Melanoma cells can elicit an immune response through the release of various cytokines, including CXCL-2, CCL-2, CSF-1, GM-CSF, A9, S100A8, and VEGFA. Some of these cytokines, such as GM-CSF and VEGFA, can stimulate the activation of macrophages, transforming these cells into TAMs. The activation of macrophages also results in the release of a series of factors, including TGF-β, CCL-22, and IL-10, which can influence dendritic cells and T-lymphocytes. In addition, TAMs can release TNF-α and interferon-γ to target cancer cells. It is worth noting that matrix metalloproteinases 9 and 2 (MMP9 and MMP2) can break down collagen in the tissue surrounding the melanoma mass, contributing to its decomposition

In a different study, the collagen-structured anti-macrophage receptor was discovered to induce TAMs polarization into pro-inflammatory phenotypes, leading to anti-tumor immunological responses in B16 melanomas [36]. Furthermore, Gordon et al. showed that suppression of PD-1/PD-L1 in vivo promoted macrophage phagocytosis, decreased tumor progression, and improved macrophage survival [16, 144]. Lymphocyte-Activation Gene 3 (LAG-3, CD223) is another important immune checkpoint molecule that participates in T-cell exhaustion similar to Cytotoxic T-Lymphocyte Antigen 4 (CTLA-4) and Programmed cell death protein 1 (PD-1) [145]. It is expressed on the surface of activated CD4^+^ and CD8^+^ T cells and other immune cells, such as natural killer cells, regulatory T cells, and macrophages [146, 147]. TAMs release chemokines that lead to the recruitment of immune-suppressive cells towards the tumor microenvironment, which influence other stromal cells, like fibroblasts, to synthesize chemokines. Young et al. demonstrated that granulocytic MDSCs are recruited to tumor sites by the CXCR2 ligand, produced by fibroblasts, after being stimulated by IL-1β from TAMs [148]. Moreover, combination therapy with anti-CD115 Abs and CXCR2 agonists might inhibit B16F10 melanoma in vivo by preventing the enrollment of granulocytic MDSCs and removing immature TAMs [149]. Notably, emactuzumab, an anti-human CD115 Ab, reduced CD163^+^ CD206^+^ M2 macrophages in melanoma cases by eliminating immature TAMs before being stimulated by IL-4 [150]. These findings imply that anti-CXCR2 agonists and emactuzumab may trigger the anti-melanoma immune response by lowering M2 polarized TAMs. These results highlight the necessity of understanding how chemotherapeutic agents affect TAMs. The ICIs, in combination with TAMs targeting agents, provide promising outcomes for melanoma treatment. Data from preclinical research provided good explanations for clinical trials in which elimination or repolarization of immunosuppressive TAMs are being investigated to overcome ICI resistance and improve their anti-tumor functions [151]. Studies using ICIs and immunomodulatory factors that block M2-TAMs activities have been performed or are still being conducted in melanoma patients. Decreases in M-CSF (CSF-1) and increases in GM-CSF levels are two strategies being investigated in conjunction with ICIs to re-polarize M2-TAMs into M1-TAMs. For example, the phase 2 studies of recombinant human analog (sargramostim) as a GM-CSF agonist in combination with ipilimumab for the treatment of unresectable stage III or IV metastatic melanoma has been completed and revealed increased survival [152–154]. Talimogene laherparepvec (T-VEC), a modified oncolytic herpes virus, is another GM-CSF agonist that increases the anti-tumor responses and has been approved for local treatment of advanced melanoma. The T-VEC exclusively infects and replicates in tumor cells and results in immune-mediated lysis of tumor cells via encoding human GM-CSF, as well as the susceptibility of melanoma to ICIs. Combination therapy of melanoma with T-VEC plus nivolumab and pembrolizumab has reached phase 2 clinical trial [155–157]. OPTiM is also a phase III trial of talimogene laherparepvec, in which T-VEC had long-term efficacy in contrast to GM-CSF in advanced melanoma [158, 159]. Moreover, ONCOS-102 is an engineered oncolytic adenovirus encoding GM-CSF that has shown synergistic effects for metastatic or unrespectable melanoma treatment in combination with pembrolizumab (anti-PD-1 Ab) [160, 161]. Designing antagonists against M-CSF cytokine is another strategy that might lead to the depletion of M2-TAMs and an improvement in ICIs functions. In addition, M-CSF contributes to metastatic melanoma resistance to BRAF-targeted therapies. Therefore, M-CSF acts as a therapeutic target in BRAFV600E melanoma. Monoclonal antibody lacnotuzumab, an anti-M-CSF, has been studied alone and in combination with ICI spartalizumab (an anti-PD-1 mAb) [162–165]. The M-CSF receptor (CSF1R) provides another therapeutic target to deplete the immunosuppressive functions of TAMs. Some examples include BLZ945 (CSF1R inhibitor) combined with PDR001 (anti-PD-1 mAb), LY3022855 (CSF1R inhibitor) combined with tremelimumab or durvalumab ICIs, emactuzumab (CSF1R inhibitor), and cabiralizumab (a humanized mAb against CSF1R) [166–170]. APX005M is a humanized CD40 agonist mAb that activates immune responses by stimulating IFN-γ secretion [170]. INCB001158 is an arginase inhibitor used as monotherapy or combined with pembrolizumab in solid metastatic tumors such as melanoma. It has been suggested that inhibition of metabolic enzymes, such as ARG-1, could restore T-cell activities by filling arginine storage [171]. Moreover, it has been reported that PI3K-γ inhibition can re-polarize M2-TAMs into pro-inflammatory M1-TAMs. Moreover, IPI-549 is a PI3K inhibitor used alone or in combination with nivolumab (Fig. 5) [172, 173].

## Macrophages in glioma cancer (GBM)

Glioma is a type of primary brain tumor, including glioblastoma, astrocytoma, and oligodendroglioma [174]. The microenvironment of glioma is characterized by the presence of macrophages and microglia, known as tumor-associated macrophages [175, 176]. Microglia, which are phagocytes of the central nervous system, exist in three forms: amoeboid, ramified, and reactive [177, 178]. Amoeboid microglia are involved in embryonic central nervous system development [179], while ramified microglia are found in large quantities in the brain parenchyma with the ability to transform into neurons, astrocytes, or oligodendrocytes [180, 181]. Reactive microglia, which are rod-like with non-branching processes and numerous lysosomes and phagosomes, are associated with brain injury and neuroinflammation [182, 183]. They also secrete MHC class II antigens and produce inflammatory mediators [184, 185]. Macrophages in the central nervous system can be classified according to their location as perivascular macrophages, meningeal macrophages, macrophages of the circumventricular organs, or macrophages of the choroid plexus. Among the brain cancers mentioned above, glioma is a particularly aggressive and untreatable type of brain tumor with a poor prognosis, and current treatments have not been successful in improving outcomes [186, 187]. Therefore, there is a need for further research into the mechanisms behind the invasiveness and recurrence of glioma and the development of new therapeutic approaches, including immunologic treatment [188, 189].

Glioma-associated macrophages (GAMs) are a key component of the tumor microenvironment in gliomas [190] that can be derived from microglia as well as bone marrow-derived macrophages [191–194]. The number and characteristics of GAMs can vary significantly, with evidence from single-cell sequencing showing that GAMs are made up of 59.05% and 27.87% of immunocytes in primary and recurrent glioblastomas, respectively [195]. Various signaling molecules, growth factors, transcription factors, and epigenetic and post-transcriptional modifications influence the phenotype and activation state of GAMs. Depending on their origin, these cells can exhibit different characteristics, with some derived from brain-resident microglia [196] and others from bone marrow-derived monocytes [197]. The GAMs play a role in various aspects of glioma progression, such as cell motility, proliferation, survival [188], and immune suppression [198, 199]. They can also produce a range of growth factors and pro-inflammatory cytokines that contribute to the supportive matrix for tumor cell metastasis and the development of an immunosuppressive microenvironment [200]. Understanding the role of GAMs in the tumor microenvironment may provide insights into potential therapeutic approaches for gliomas. In this context, Woolf et al. demonstrated using single-cell imaging that P2RY12 and TMEM119 label microglia in GBM, and they further demonstrated that these markers could be used to distinguish microglia from BMDM. P2RY12 protein expression is associated with longer survival rates in patients. Activation of P2Y12 receptors has been linked to the extension of microglial cell processes [201, 202]. Moreover, another study that analyzed marker genes in GAMs found that only a small number of genes were consistently present, indicating the diverse responses observed in different settings. In this regard, Tgm2 and Gpnmb genes were the only ones that were common across the analyzed data sets, highlighting the need for further research to understand the functional state of GAMs.

### GAMs regulating GBM malignancy

In the presence of glioblastoma (GBM) cells, the functions of microglia may be impaired, leading to the initiation or growth of tumors. This has been demonstrated through comparative transcriptome analysis. It was found that GBM-bearing mice's microglia are less sensitive and impaired at monitoring immunity due to a reduction in a group of genes that encode receptors for various antigens, chemokines, and cytokines [203]. Additionally, microglia engage in reciprocal molecular crosstalk with glioblastoma stem cells, exhibiting a more direct pro-tumorigenic function through the secretion of TGF-β [204]. Microglia activated by GM-CSF can release CCL5, a chemokine that upregulates the secretion of MMP2 in GBM cells, thereby promoting tumor migration and invasion [205]. This effect may be mediated by the secretion of interferon-gamma (IFNγ) by infiltrating microglia, which leads to the stable expression of a specific transcriptional program in GBM cells that is associated with myeloid cells [206]. This epigenetic immunoediting may also be present in human mesenchymal subtype glioblastoma stem cells (GSCs) [207]. The TAMs also play a role in GBM invasion through the expression of CCL8 and the activation of signaling pathways in GBM cells through the binding of CCL8 to CCR1 and CCR5 receptors [208], the secretion of CSF-1 [209] and epidermal growth factor (EGF) by GBM and microglia, respectively, have also been shown to stimulate GBM invasion through the recruitment of TAMs and activation of signaling pathways in GBM cells through the binding of EGF to epidermal growth factor receptors (EGFR) [210].

### GAMs in angiogenesis of GBM

**Fig. 6:**
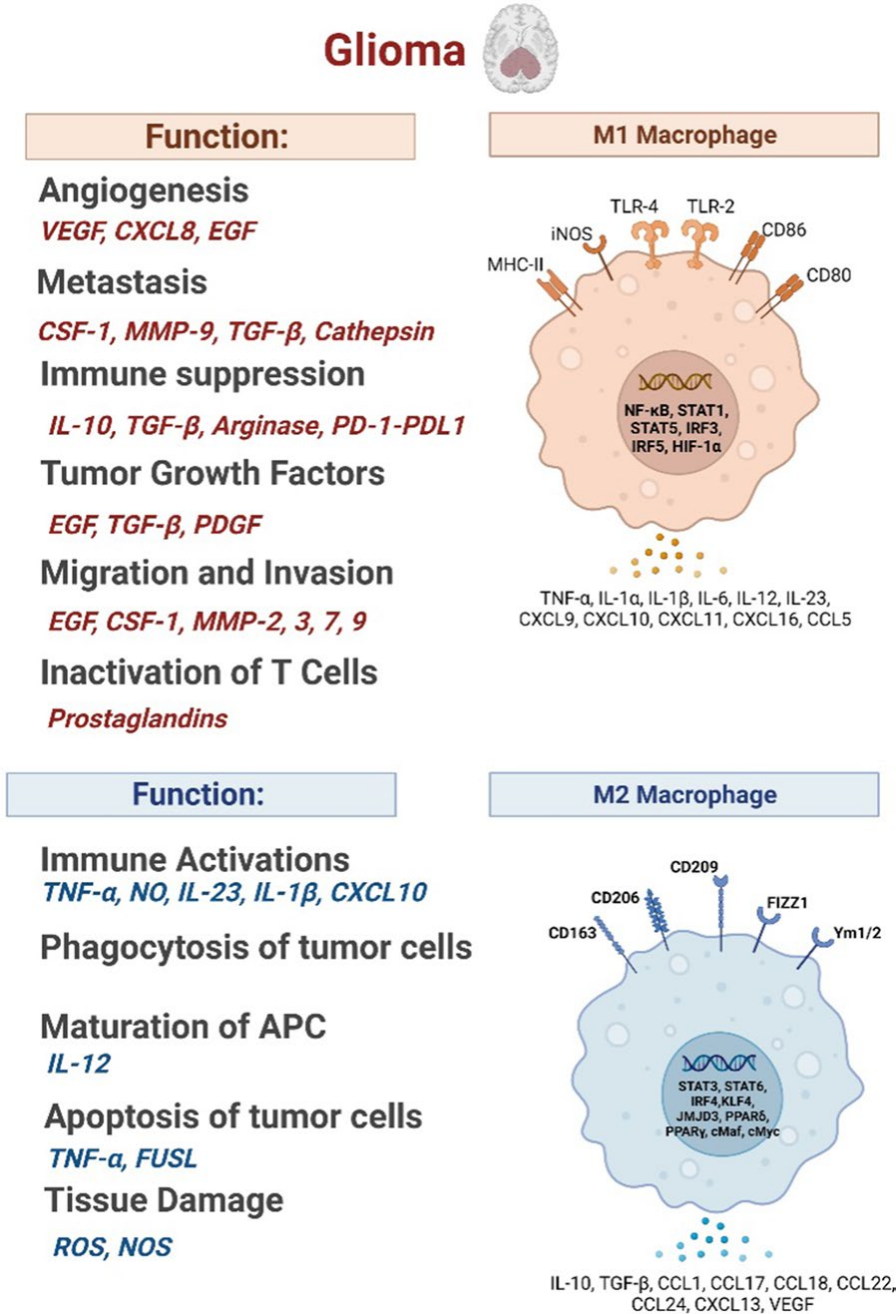
Anti-tumor/pro-tumor activity of macrophages in GBM

The resistance of GBM to anti-VEGF therapy, which targets a protein involved in angiogenesis, has been linked to the macrophages infiltration into the tumor (Fig. 6) [211]. It depends on the activation state of the immune cells and whether they promote or suppress angiogenesis. Immunosuppressive macrophages like M2 promote angiogenesis, while pro-inflammatory macrophages like M1 suppress it [212]. Depletion of TAMs in animal models has been shown to reduce the blood vessel density in GBM, suggesting a role of these cells in GBM angiogenesis [213]. Resident microglia may be particularly important in this process, as their selective depletion has been shown to reduce blood vessels in GBM to a greater extent than the depletion of all TAMs [213]. The TAMs isolated from a specific type of glioma have been found to overexpress proangiogenic factors such as VEGF and CXCL2, both of which have been linked to angiogenesis. The interaction of the receptor for advanced glycation end products (RAGE) with its ligands has also been shown to promote angiogenesis in GBM through the activation of TAMs-specific signaling pathways [214].

### GAMs in drug resistance of GBM

Resistance to temozolomide (TMZ) has been reported as a common obstacle to GBM patients’ treatment, where the resistance rate is approximately 60% [215]. Literature evidence suggests that genetic factors and GAMs may contribute to this resistance [216]. The interleukin-11 (IL-11) produced by microglia and macrophages activates STAT3-MYC signaling in GBM cells, leading to TMZ resistance [216]. By inhibiting GAM recruitment and IL-11 secretion through ABP1 ablation or genetic inactivation, TMZ resistance has been reversed in a murine model of GBM [217]. Additionally, different subpopulations of GAMs may have distinct effects on treatment responses [218]. For instance, M2-like GAMs contribute to resistance through secretion of exosomal miR-21-5p, while M1-like polarization of GAMs induced by GBM-derived extracellular HMGB1 has been shown to restore sensitivity to TMZ. In addition to chemoresistance, GAMs have also been implicated in resistance to radiotherapy and antiangiogenic therapy [219]. The impact of GAMs on treatment responses may be mediated by the expression of PD-L1, which interacts with CD80 on T-cells and leads to CD4^+^ T-cell suppression, Treg expansion, and immune checkpoint blockade resistance [220]. The role of CD73-expressing macrophages in ICB resistance has also been demonstrated in a murine model of GBM [221].

### GAM-targeted therapy in GBM

Several approaches have been identified and tested in experimental and clinical settings for targeting TAMs in glioblastoma (GBM). These approaches can be divided into three categories: TAM re-education, TAM education, and TAM depletion. TAM education involves activating pro-inflammatory pathways, which can also be delivered through gene therapy or direct administration, while TAM depletion involves targeting key molecules to achieve the unbiased depletion of TAMs or to inhibit macrophage infiltration. These TAM-targeting strategies can potentially counter immunotherapies and influence glioma progression [174].

#### Anti-angiogenic treatment

Tumor-infiltrating myeloid cells may play a role in the limited effectiveness of anti-angiogenic therapies by expressing alternative proangiogenic factors that bypass VEGF-mediated pathways [222]. The MerTK inhibitor MRX-2843 has been shown to have therapeutic benefits by promoting the polarization of macrophages away from immunosuppressive conditions, inhibiting neo-angiogenesis in the glioblastoma microenvironment, and inducing tumor cell death [223]. The metalloprotease-disintegrin ADAM8, which is highly expressed in tumor cells and associated immune cells in glioblastomas, is related to angiogenesis and is associated with a poor clinical prognosis [224]. The regulation of osteopontin mediates the angiogenic potential of ADAM8 in glioblastoma cells/primary macrophages, so targeting ADAM8 may be a viable approach for modulating angiogenesis in glioblastoma [225].

#### PD-L1 signaling pathway

Pembrolizumab monotherapy, which targets the PD-1 protein, cannot elicit an effective immune response in most GBM patients, likely due to the low number of T cells within the tumor microenvironment and the abundance of CD68 + macrophages [226]. Besides that, in a recent study, it was reported that GBM cells secrete interleukin-11 (IL11) in response to glial-derived neurotrophic GAMs, activating signal transducer and activator of transcription 3-MYC signaling. This signaling pathway leads to the induction of stem cell states, which increase tumorigenicity and resistance to temozolomide (TMZ) in GBM cells. In mouse GBM models, PI3K inactivation or inhibition reduces microglia recruitment and IL11 secretion, resulting in improved TMZ response [227]. Anyway**,** in the late stage of temozolomide (TMZ) treatment or relapse, treatment with an anti-PD-L1 antibody significantly reduced the infiltration of CD163-positive macrophages into tumors. In contrast, a combination of a PD-L1 antibody and IPI-549 (a selective PI3Kγ inhibitor) therapy effectively inhibited tumor growth [228]. Treatment with rapamycin and hydroxychloroquine (RQ) decreased the polarization of M2 macrophages, increased phagocytic ability, and increased the accumulation of lipid droplets. This treatment enhanced the ratio of anti-tumoral to pro-tumoral immune cells within the tumor and the ratio of CD8 to CD4 T cells. The combination of RQ and anti-PD1 treatment was found to be synergistic in action [229]. Saha et al. tested a triple combination of anti-CTLA-4, anti-PD-1, and G47Δ-mIL12 (oncolytic herpes simplex viruses armed with angiostatin and IL-12) in mouse GBM models. This treatment was associated with an influx of macrophages, an anti-tumoral, macrophage-like polarization of these cells, and an increase in the ratio of T-effector to T regulatory cells. This treatment was able to cure most mice with gliomas. Immune cell depletion studies showed that CD4^+^ and CD8^+^ T cells and macrophages are all required for the synergistic curative activity of this treatment [230].

#### Combination therapy

Several studies have reported the potential of targeting pro-tumoral macrophages in the treatment of GBM. Almahariq et al. found that the BLZ-945, a CSF-1R inhibitor, reduced pro-tumoral macrophage polarization and improved the response to radiotherapy in respected tumors with a high baseline population of pro-tumoral macrophages [231]. The results of Zhu et al. showed that when debulking plus anti-CD47 tumors were compared with non-debulking plus IgG tumors, macrophages with CD68-positive labels were recruited more, pro-inflammatory cytokines like CXCL10 were increased, and angiogenic proteins were decreased, indicating that surgical resections coupled with anti-CD47 blocking immunotherapy promote inflammation and prolong survival [232]. As a result of lipopolysaccharide and interferon-gamma stimulation of bone marrow macrophages and brain-resident macrophages, Herting et al. have found that dexamethasone prevented the production of IL-1. These findings suggest that IL-1 signaling may be a useful therapeutic target in the management of GBM-associated cerebral edema [233] (Table 1).

**Table 1:** Macrophages-based therapeutic strategies in four different cancers: breast, glioma, colorectal, and melanoma

Mechanisms of TAMs targeting agents	Cancer type	Agent Name	Function	References
GM-CSF agonist	Melanoma	1-T-VEC 2-ONCOS-102 3-Sargramostim	Induction of cytotoxic T-cell responses, immune-mediated tumor cell death, repolarization of M2-TAMs to tumor suppressive phenotype M1-TAMs	[152–154]
	Breast cancer	1-T-VEC		[234]
	Glioma	1-T-VEC2-Sargramostim		[230, 235]
	Colorectal	1-ONCOS-102		[236, 237]
M-CSF antagonist	Melanoma	1-Lacnotuzumab	Antagonists against M-CSF cytokine or its receptor (M-CSF-R) lead to the depletion of M2-TAMs and its tumor progression support, interfering with M-CSF signaling, TAM recruitment, and polarization	[162, 163, 165, 238]
	Breast cancer	1-BLZ945 2-RG7155		[239, 240]
	Glioma	1-GW2580 2-BLZ945 3-AFS98		[241–244]
	Colorectal	1-PLX3397		[245]
CSF1R antagonist	Melanoma	1-BLZ945 2-LY3022855 3-Emactuzumab 4-Cabiralizumab 5-PLX647 6-siCD115	Targeting M-CSF receptors (CSF1R) on MDSCs results in the preferential expression of M1-TAMs and inhibition of tumor growth, by modulating the TILs profiles Inhibiting the c-kit tyrosine kinase and colony-stimulating factor-1 (CSF-1) receptor kinase	[132, 166–170]
	Breast cancer	1-Pexidartinib (PLX3397)		[246]
	Glioma	1-Pexidartinib (PLX3397)		[246]
	Colorectal	1-Cabiralizumab		[247]
CD40 Agonist	Melanoma	1-APX005M	CD40 agonist, after binding to CD40, activates immune responses by stimulating the secretion of IFN-γ. Following the interaction of IFN-γ with its receptor on melanoma cells, the JAK/STAT/IRF1 downstream cascade is triggered	[170]
	Breast cancer	1-ADC-1013 2-Selicrelumab 3-ChiLob7/4 4-SEA-CD40		[248–252]
	Glioma	1-APX005M 2-6-2141-V11		[253, 254]
	Colorectal	1-ADC-1013 2-RO7009789 3-ChiLob 7/4 4-APX005M		[248, 255]
IDO inhibitor	Melanoma	1-Indoximod 2-Epacadostat (or ECHO-204)	Attenuating immune suppression in tumors through inhibition of tryptophan metabolism, inducing tumor regression by stimulating T-cell recruitment and preventing resistance to ICIs and TAMs-mediated immune evasion	[25, 256, 257]
	Breast cancer	1-NLG919 (Navoximod) 2-KHK2455 3-LY3381916		[258–262]
	Glioma	1-Indoximod 2-NLG2105 3-PF-06840003		[263, 264]
	Colorectal	1-Epacadostat 2-NLG919 (Navoximod)		[265–267]
ARG-1 inhibitor	Melanoma	1-INCB001158 (or CB-1158) 2-Piceatannol	Restore T-cell activities through replacing arginine storage, Attenuating tumor growth and mortality rate	[171, 268, 269]
	Breast cancer	1-nor-NOHA 2-CB-1158 3-Piceatannol 4-Chlorogenic acid		[269–272]
	Glioma	1-nor-NOHA 2-OATD-02 (OAT-1746)		[273, 274]
	Colorectal	1-Piceatannol 2-Chlorogenic acid		[271, 275]
PI3K inhibitor	Melanoma	1-IPI-549 2-GSK2636771	Re-polarization of pro-tumor M2-TAMs towards pro-inflammatory M1-TAMs, overexpression of IFN-γ-responsive factors, and elimination of the tumor suppressor PTEN gene	[172, 276]
	Breast cancer	1-Alpelisib (BYL719) 2-GDC-0077 (Inavolisib) 3-GDC-0941 (Pictilisib)		[277–280]
	Glioma	1-PX-866 2-LY294002 3-BKM120		[281–283]
	Colorectal	1-PX-866 2-BKM120 (Buparlisib) 3-HS-173 4-BEZ235 5-NVP-BEZ235		[284–288]
Anti-LAG-3	Melanoma	1-BMS-986016 (Relatlimab) 2-BI 754111	Inhibiting LAG-3 binding to MHC-II, increasing CD8^+^ IFNγ producing cells and decreasing tumor progression	[289, 290]
	Breast cancer	1-IMP321 (Eftilagimod alpha) 2-REGN3767		[291–293]
	Glioma	1-BMS-986016 (relatlimab)		[294, 295]
	Colorectal	1-MK4280 (favezelimab) 2-MGD013 (tebotelimab)		[296–298]

## Conclusion

The use of cancer immunotherapy for removing residual tumors has emerged as an effective way to improve the survival of patients with advanced-stage cancers, as it enhances the immune system's ability to eliminate minimal residual tumors. As a result of ineffective immune cells against cancer cells, patients with cancer are more likely to develop tumors, which reduces the effectiveness of therapeutic measures. The macrophage is one of the most important innate system cells contributing to normal homeostasis, inflammation, and phagocytosis. Several studies have shown, however, that macrophages promote genetic instability and angiogenesis in the development of oncogenesis and neoplasms. The M2 macrophages promote tumor growth and metastasis. Among the most diverse immune cells in the TME are the M2 macrophages, which along with the M1 macrophages, are called TAMs. The pro-tumorigenic M2 macrophages are attracted to tumor cells by chemokines and growth factors. Therefore, immunotherapy efficacy is also strongly influenced by changes in macrophage subpopulations. The TAMs have been implicated as a therapeutic target in numerous biological studies due to their ability to deplete, inhibit recruitment, and influence polarization status. In addition, TAMs limit the efficacy of immunotherapy approaches, such as anti-PD1 treatment, because they are linked to resistance to well-known antitumor therapies, such as chemotherapy and radiotherapy. Anyway, many preclinical studies using small molecules or antibodies to block each of mentioned factors/pathways individually have demonstrated significant improvement in response to a wide variety of tumors to therapy, indicating that their blockage is generally well tolerated. However, more research is needed to overcome macrophage-based cancer therapy, particularly in nanoparticles and drug delivery. In this line, the use of small molecules or antibodies to block specific factors or pathways associated with TAMs has shown promising results in preclinical studies, leading to improved responses to a wide variety of tumors. These approaches have generally been well tolerated. However, more research is needed, especially in the field of nanoparticles and drug delivery, to advance macrophage-based cancer therapy further. As the role of TAMs in cancer therapy is increasingly recognized, several crucial gaps in the field necessitate further investigation. TAM heterogeneity, plasticity, and their interactions with other immune cells remain areas of exploration. Understanding the underlying mechanisms of TAM-mediated immunosuppression and identifying reliable biomarkers for patient stratification and treatment response assessment is paramount. Additionally, optimizing TAM-targeted therapies and validating their clinical effectiveness are essential for translating preclinical findings into meaningful treatments.

## Data Availability

Not applicable.

## Abbreviations

TME: Tumor microenvironment
TAMs: Tumor-associated macrophages
IBC: Inflammatory breast cancer
DCIS: Ductal carcinoma in situ
IDC: Invasive ductal carcinoma
CSCs: Cancer stem cells
EMT: Epithelial–mesenchymal transition
MDSCs: Myeloid-derived suppressor cells
CSF1/CSF1R: Colony stimulating factor 1
Gpr132: G Protein-Coupled Receptor 132
HIFs: Hypoxia-inducible factors
TNFα: Tumor necrosis factor-alpha
TGFβ1: Transforming growth factor beta 1
SIGLEC1: Sialic acid-binding Ig-like lectin 1
G-CSF: Granulocyte colony-stimulating factor
MCP1: Monocyte chemotactic protein-1
MIP-1 and MIP-2: Macrophage inflammatory proteins-1 and -2
MK2: MAPK-activated protein kinase 2
CRC: Colorectal cancer
lncRNA: Long non-coding RNAs
TICs: Tumor-initiating cells
MTF: Tumor cell fusion
Tregs: Regulatory T cells
MDSCs: Myeloid-derived suppressor cells
MARCO: Macrophage receptor with collagenous structure
GBM: Glioblastoma
